# Interpreting finite element results for brittle materials in endodontic restorations

**DOI:** 10.1186/1475-925X-10-44

**Published:** 2011-06-02

**Authors:** Antonio Pérez-González, José L Iserte-Vilar, Carmen González-Lluch

**Affiliations:** 1 Department of Mechanical Engineering and Construction, Universitat Jaume I, Castellón de la Plana, Spain

## Abstract

**Background:**

Finite element simulation has been used in last years for analysing the biomechanical performance of post-core restorations in endodontics, but results of these simulations have been interpreted in most of the works using von Mises stress criterion. However, the validity of this failure criterion for brittle materials, which are present in these restorations, is questionable. The objective of the paper is to analyse how finite element results for brittle materials of endodontic restorations should be interpreted to obtain correct conclusions about the possible failure in the restoration.

**Methods:**

Different failure criteria (Von Mises, Rankine, Coulomb-Mohr, Modified Mohr and Christensen) and material strength data (diametral tensile strength and flexural strength) were considered in the study. Three finite element models (FEM) were developed to simulate an endodontic restoration and two typical material tests: diametral tensile test and flexural test.

**Results:**

Results showed that the Christensen criterion predicts similar results as the Von Mises criterion for ductile components, while it predicts similar results to all other criteria for brittle components. The different criteria predict different failure points for the diametral tensile test, all of them under multi-axial stress states. All criteria except Von Mises predict failure for flexural test at the same point of the specimen, with this point under uniaxial tensile stress.

**Conclusions:**

From the results it is concluded that the Christensen criterion is recommended for FEM result interpretation in endodontic restorations and that the flexural test is recommended to estimate tensile strength instead of the diametral tensile test.

## Background

Advances in endodontic restoration in dentistry have generalized the use of prefabricated posts for restoring devitalized teeth, especially when the coronal tooth structure is severely damaged [1]. The artificial post connects to the core over which is placed the restored crown, helping the transmission of the dental loads from the coronal structure to the root.

In recent decades, finite element (FE) simulation has been increasingly employed for analysing the biomechanics of the post-core endodontic restorations [2-13]. FE simulation has the advantage that random variability is avoided, unlike experimental in vitro tests. Moreover, the results obtained from a finite element model (FEM) of the restored system contain information about the stress distribution of each component of the restoration, instead of only a single value of failure load typical of in vitro results.

A correct interpretation of FEM results should be based on the stresses and strength of each component in the system. To obtain accurate conclusions from this interpretation, three conditions must be fulfilled: first, stress values must be reliable, i.e., the FEM should adequately represent the real system; second, strengths of the different materials present in the model must be known; third, an adequate failure criterion must be used to compare values of computed stresses, which are bi-axial or tri-axial, with material strengths frequently obtained under conditions of uniaxial stress state (tension or compression). The first condition is progressively approached with three-dimensional models, with finer meshes and more components represented in the system, although a good representation of bonded interfaces is difficult due to its small thickness. Moreover, some uncertainty exists about some of the material elastic properties. In addition, for most models all materials are considered to have linear isotropic behaviour, though this is a simplification for components such as dentine or fibre posts. However, the second and third conditions are not met in most previous work: the second due to the lack of consistent and complete data about the strength of some dental materials; the third due to the absence of a post-hoc analysis of failure in each component of the FEM or the use of the Von Mises equivalent stress as the reference value for this analysis. It is known that the Von Mises criterion is only valid for ductile materials with equal compressive and tensile strength [14], but materials exhibiting brittle behaviour such as ceramics, cements or resin composites are used frequently is these restorations. Even dentine presents reported values of compressive strength significantly greater than tensile strength [15].

Little research has been devoted to the interpretation of FEM results in post-core restorations. Most previous work has analysed the results of FE simulations from Von Mises maximal stresses [4-6,8,9,11-13,16]. The prevalent use of the Von Mises criterion is probably associated to the fact that this is the normal criterion for most engineering analyses, which usually deal with ductile materials such as steel or aluminium. All the commercial FE programs include this criterion as one of the default outputs in their post-processing modules. Moreover, this criterion makes it possible to talk in terms of an equivalent Von Mises stress, which is directly comparable to tensile strength and is thus a nice, simple failure criterion. For brittle materials, however, it is not possible to obtain an equivalent stress from the stress tensor, regardless of the material strengths, so that it can be compared with a strength value, because the compressive strength is normally different and greater than the tensile strength. To make up for this, some authors suggest the use of the Rankine or Maximum Normal Stress criterion to evaluate the failure in dentine, using the maximum principal stress to analyse the results [17,18]. Others analyse results of shear stress in the post-dentine interface, indicating that this value should be compared to the reported shear bond strengths to evaluate the risk losing retention [4,5]. Dejak et al. [19] applied the Tsai-Wu criterion for anisotropic materials to dentine, enamel and resin composites in molars with ceramic inlays. DeGroot et al. [14] compared three criteria to analyse FEM results in composite resin: Von Mises, a modified Von Mises criterion presented by Williams [20], and the Drüker-Prager criterion, concluding that Drüker-Prager is more suitable to describe the failure of this material. Recently, Christensen [21] proposed a unified failure criterion for ductile and brittle materials, which is equivalent to the modified Von Mises criterion proposed by Williams with an additional modification for brittle materials, and demonstrated some unrealistic behaviour of the Drüker-Prager and Coulomb-Mohr criteria under some important stress states.

Regardless of the failure criterion, reliable information about compressive and tensile strengths is needed for each material in the restoration. The compressive strength is usually obtained experimentally by a compressive test using cylindrical specimens. Tensile strength is obtained by applying an axial pulling force over specimens with a cylindrical or rectangular cross section and is a typical test for metals and other ductile materials. This type of test, however, is rarely used for brittle materials. Technical problems related with gripping and aligning the brittle specimens are often cited as an explanation for not measuring the tensile strengths [22,23]. Alternatively, the diametral tensile test (DTT) is commonly used to obtain a diametral tensile strength (DTS) [23-26] because of its simplicity and reproducibility [27]. The DTT is performed by compressing a cylindrical specimen with its axis being perpendicular to the load direction. Tensile strength can also be obtained indirectly as a flexural strength (FS) with three or four point flexural tests (FT) [23,25]. However, DTS and FS are obtained in loading states that are not uniaxial and the results of these tests are not equivalent, as numerous previous works have shown for different dental materials [22,23,25,28,29]. Despite this, DTS and FS, have been used interchangeably in recent works as a reference to compare to computed maximal stresses in finite element models of dental restorations [30,31].

The objective of this paper is to discuss the problem of interpreting finite element results of the simulation of dental restorations and to propose some rules to do this interpretation correctly, with special attention to the failure criteria in brittle components. Additionally, the relationship between DTS and FS is analysed and the procedure for the correct use of these values for the failure criteria of brittle materials is discussed.

## Methods

Different failure theories employed in mechanical analyses were compared in this study to determine how suitable they were for use during the interpretation of FE results in dental restorations. These theories were: Von Mises (VM), Rankine (R), Coulomb-Mohr (CM), Modified Mohr (MM) and Christensen (C). All of these theories combine principal stresses at a point in a solid (*σ*_1_, *σ*_2_, *σ*_3_) with the compressive strength (CS) and tensile strength (TS) of the material to obtain a safety factor (SF) at this point. Safety factor values lower than unity indicate that the material is prone to have a mechanical failure at this point, and values greater than unity indicate a safe condition at this point. All of these criteria are used in mechanical engineering texts [32], except the Christensen criterion, which has only been recently presented in the literature [21]. These criteria are formulated below, with *σ*_1 _≥ *σ*_2 _≥ *σ*_3 _and *CS *≥ 0, *TS *≥ 0.

The Von Mises stress criterion is used for ductile materials considering only TS in its formulation because in most ductile materials TS is similar to CS, and is expressed as:(1)

where *J_2 _*is the second invariant of the deviatoric stress tensor:(2)

The rest of criteria use the two mechanical properties, TS and CS, in their formulation. Rankine stress criterion can be expressed as:(3)

The Coulomb-Mohr criterion is used for brittle materials where the maximum principal stress is positive (*σ*_1 _≥ 0) and the minimal principal stress is negative (*σ*_3 _≤ 0), whereas the Rankine criterion is used in other situations. The Coulomb-Mohr criterion is formulated as:(4)

where *k *represents the ratio between the compressive and tensile strength of the material:(5)

The Modified Mohr criterion includes a modification of the Coulomb-Mohr theory to better fit experimental data and is expressed as:(6)

The Christensen criterion, recently presented in the literature [21], is valid only for *CS *>*TS*. After some algebraic manipulation, the safety factor for this criterion can be written, for ductile materials (considered ductile if *TS *>*CS*/2), as (see [14]):(7)

where *I_1 _*is the first invariant of the stress tensor:(8)

The expression in Eq. 7 reduces to the Von Mises criterion for materials with TS equal to CS (*k *= 1). For brittle materials (*TS *≤ *CS*/2), the Christensen criterion includes an additional condition for failure under tensile state:(9)

To compare these criteria, a hypothetical brittle material with TS = 100 MPa and CS = 300 MPa, which is in the range of some brittle dental materials, was considered and the different criteria were compared in a plane stress state.

Three different finite element models were developed in this work to analyse the effect of failure criteria in the FEM results interpretation and to compare DTT and FT for obtaining material properties to be used for these criteria. The CosmosWorks module of the SolidWorks CAD/CAE system (Dassault Systèmes SolidWorks Corp., Concord, MA, USA) and Nastran (MSC.Software Corporation, Santa Ana, CA, USA), were used to solve the models.

**Figure 1:**
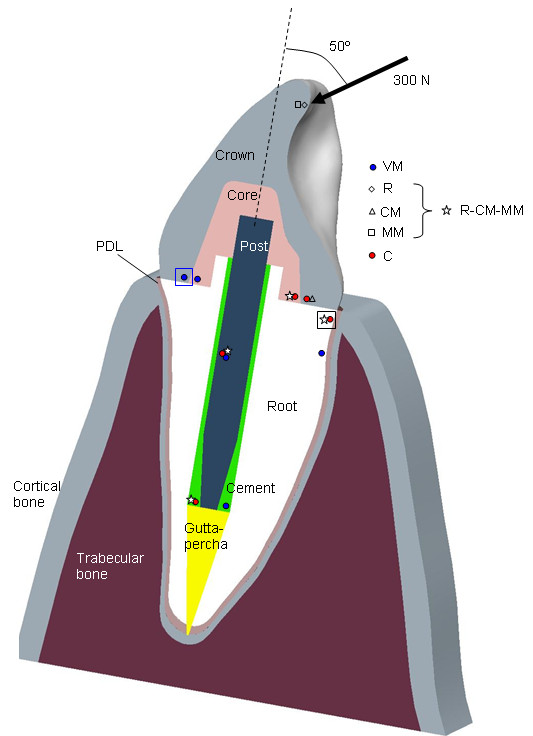
**Section of the tooth model with position of failure initiation points for the different criteria**. Longitudinal section of the geometrical model simulating a typical endodontic restoration of a maxillary central incisor including the elements: bone (cortical and trabecular components), periodontal ligament (PDL), root, gutta-percha, post, cement, core and crown. Position and orientation of the load applied for the FE model to simulate a real biting force. Marks indicate position of the points with the lowest SF in each component for the different criteria considered. A common mark was used for R, CM and MM criteria when they predicted the critical point in the same position. Big squares indicate the area predicted for failure initiation by the different criteria (VM: blue square, R-CM-MM-C: black square).

The first FE model simulated a typical endodontic restoration of a maxillary central incisor. The model was based on the geometry of a real maxillary central incisor obtained by means of a 3D scanner. Pro ⁄ Engineer CAD system (PTC, Needham, MA, USA) was used to generate, and later assemble, the geometries for all the components included in the model. Figure 1 shows a longitudinal section of the geometrical model, including all the components that were modelled, namely bone (cortical and trabecular components), periodontal ligament (PDL), root, gutta-percha, post, cement, core, and crown. The model was prepared with two different typical configurations in the coronal dentine (one using a ferrule with a height of 1.5 mm and a diameter of 3 mm and the other without a ferrule) to consider the possible effect of this factor. The mechanical properties of the different components of the model were taken from the literature and manufacturer data and are presented in Table 1. The Pro ⁄ Mechanica module, available within Pro ⁄ Engineer, was used to generate a FE mesh from CAD geometry. Solid tetrahedral elements were created with a mesh control to limit the maximal size of the elements to 0.3 mm for all the components, except for the trabecular and cortical bone where a maximal size of 1 mm was considered. The final model had almost 399,000 elements defined by approximately 69,000 nodes. As boundary conditions, the displacements in the medial distal direction of all the nodes on the lateral surface and all the displacements of the base of the components representing the bone were restricted. A 300 N load was distributed over a small area of 8 mm^2 ^on the palatal side of the tooth, near the incisal edge. This load was applied with an angle of 50° to the radicular axis, in the vestibular direction, as shown in Figure 1, to simulate real biting forces. The analysis was carried out using the finite element analysis software application MSC-Patran-Nastran. Similar models of endodontic restorations were used by the authors in previous works [7,12] with results that agreed well with the experimental data, thus confirming the fidelity of the model. Once the results of this model had been obtained, different failure criteria (VM, R, CM, MM, C) were applied to every element in the model to obtain the safety factor and the failure point predicted with each criterion. Table 2 shows the TS and CS considered for the different materials.

**Table 1:** Material properties of the restored tooth model

Component (Material)	Elastic Modulus (GPa)	Poisson coefficient	Reference
Root (dentine)	18.6	0.31	[3-5,8]
Gutta-percha	0.00069	0.45	[3-5]
Periodontal ligament	0.0689	0.45	[5,8]
Cortical bone	13.7	0.30	[3-5,38]
Trabecular bone	1.37	0.30	[3-5,38]
Post cement (resin cement)	18.6	0.30	Coltène Whaledent
Core (resin composite)	7.786	0.30	[34]
Crown (porcelain)	120	0.28	[4]
Post (stainless steel)	207	0.30	Coltène Whaledent

**Table 2:** CS and TS of materials, used for failure theories

Component (Material)	TS (MPa)	CS (MPa)	Reference
Root (dentine)	106	297	[15]
Post cement (resin cement)	106	242	Coltène Whaledent
Core (resin composite)	90	230	[34]
Crown (porcelain)	121^(^*^)^	162	[25]
Post (stainless steel)	1436	1436	[39]

^(*) ^estimated

**Figure 2:**
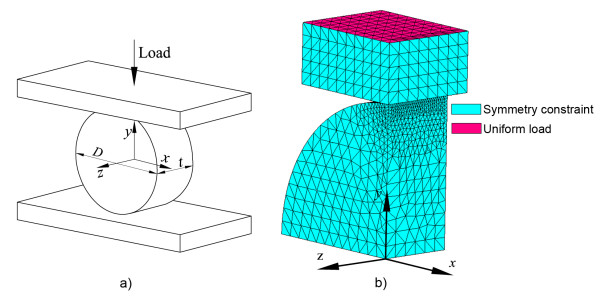
**Diametral tensile test (DTT)**. Geometry and parameters of a typical diametral tensile test on a cylindrical specimen of a brittle material (a), and its corresponding FE model with three planes of symmetry (b).

The second model simulates a typical diametral tensile test (see Figure 2a) over a cylindrical specimen of a brittle material. A diameter of D = 6 mm, typical for this test, was considered for the specimen. Several models with different specimen thicknesses (*t*) were analysed to study the effect of the diameter-to-thickness ratio, which was varied in previous works [23,29,33]. The material properties used for the specimen in the model simulate properties of ParaCore composite resin [34]: elastic modulus E = 7786 MPa and Poisson's ratio ν = 0.3. For the upper and lower compression platens the material properties of steel were considered: E = 206GPa, ν = 0.29.

For the FE meshing, the three symmetry planes of the model were applied (neglecting the force of gravity) considering only 1/8^th ^of the cylindrical specimen and adding the consequent symmetry constraints (see Figure 2b). The mesh in the specimen was refined progressively towards the contact area. The load, P, was uniformly distributed on the upper plane of the compression platen and a contact condition between the compression platen and the disc was included in the model. The magnitude of the load P in each model was selected as a function of disc thickness (Table 3) to obtain a DTS of 45 MPa, a value in the range of the reported strength of ParaCore resin composite [34,35]. The DTS value is calculated as the tensile stress in the x-direction in the disc diameter given by x = 0, z = 0, assuming plane stress and a point load [36]:(10)

**Table 3:** Load values for DTT models as a function of disc thickness

t (mm)	1	2	3	4	5	6
Load (N)	424	848	1272	1696	2120	2544

where P is the failure load and *t *the disc thickness.

**Figure 3:**
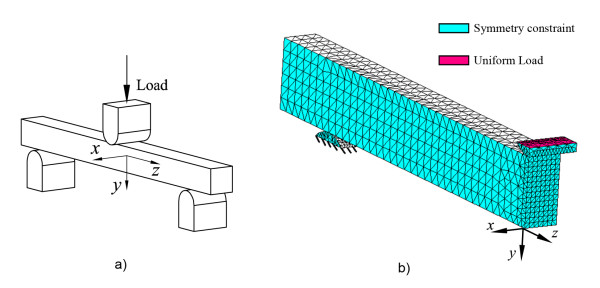
**Three-point flexural test (FT)**. Geometry of a typical three-point flexural test (a) and its corresponding FE mesh with two planes of symmetry (b).

A third FE model was created for simulating a typical three-point flexural test (see Figure 3a). A prismatic specimen of 2 × 2 × 25 mm was considered, according to ISO 4049 and a distance of 20 mm was used between supports. The same material properties of ParaCore were considered for the specimen with steel for the anvils of load application and support. The load applied in this case was P = 6 N in order to obtain a FS of 90 MPa, typical for ParaCore [34]. FS in a three-point flexural test is calculated with:(11)

where L is the distance between supports, and B and H the width and height of the specimen section, respectively.

Figure 3b shows the mesh of the FEM representing only a quarter of the specimen, taking advantage of the inherent symmetries. A refined mesh was used near the centre of the specimen. Contact conditions between the anvils and the specimen were used in the model.

FE models for DTT and FT were meshed and solved using CosmosWorks.

## Results

**Figure 4:**
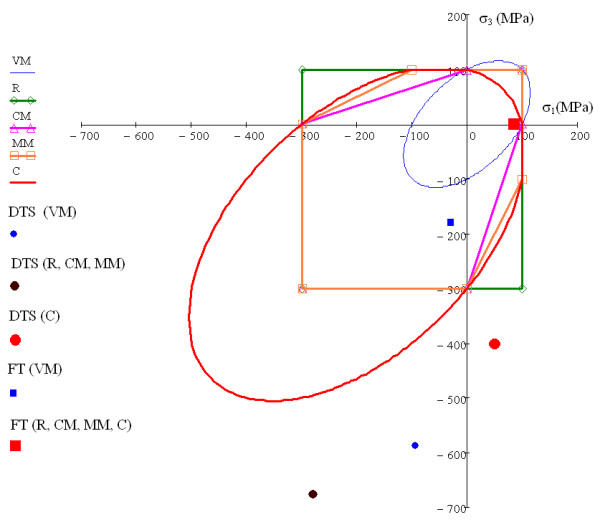
**Failure lines for different criteria and location of critical points for DTT and FT**. Failure lines for the different failure criteria in a planar stress condition (*σ_2 _*= 0) in a plot of maximum principal stress *σ_1 _*against minimum principal stress *σ_3_*, for a hypothetical brittle material with TS = 100 MPa and CS = 300 MPa, together with the location of the critical points for DTT and FT with the different failure criteria.

Figure 4 shows the failure lines for the different failure criteria in a planar stress condition (*σ_2 _*= 0) in a plot of maximum principal stress *σ_1 _*against minimum principal stress *σ_3_*. Points inside the closed line of each criterion are safe points and points outside the line will fail under this load combination for the corresponding criterion. The VM criterion, typical for ductile materials, presents quite different results compared to other criteria, especially with negative principal stresses (3rd quadrant of the plot). Typical criteria for brittle materials, such as CM and MM, are equivalent in the 1st and 3rd quadrant and similar in the 4th quadrant. The C criterion, used for a brittle material (Eq. 9), is very similar to the MM criterion in the 4th quadrant, but quite different in the 1st and 3rd ones, being more restrictive for positive principal stresses (tension) and less for negative principal stresses (compression). From the comparison, it is clear that in brittle materials, with CS greater than TS, the VM criterion would produce very different results to CM or MM.

Table 4 shows SF and principal stresses at the most critical point for each component of the restored tooth model with a ferrule, obtained with different failure criteria. All of the criteria for brittle components (R, CM, MM) predict similar results and failure initiation at dentine (lowest SF). In contrast, the VM criterion predicts failure initiation at the crown. The C criterion agrees with VM in the SF and failure point for the post, which is made of stainless steel, a ductile material, and gives similar results to R, CM and MM for the rest of components, which are brittle, with the exception of the crown. Similar conclusions were obtained for the model without a ferrule, being the failure initiation point in the same component for each of the failure criteria. Some changes were registered in the SF of certain components when the ferrule was absent. The component most affected by this change was the core, where the SF decreased between 13% and 22%, depending on the failure criteria used; the changes in the SF for other components, however, were small and always below 10%.

**Table 4:** Safety Factor and principal stresses (in brackets, MPa) at the most critical point for each component

	Cement	Dentine	Post	Crown	Core
VM	3.96 (-2.3,-4.9,-30.2)	2.00 (57.6,7.2,4.0)	8.98 (2.0,-7.2,-161.2)	1.69 (-4.6,-12.2,-80.8)	7.76 (-3.0,-4.9,-15.2)

R	4.30 (24.6,10.8,1.3)	1.55 (66.9,23.4,20.1)	8.71 (-3.2,-10.7,-163.9)	1.79 (-10.6,-39.6,-91.3)	8.65 (10.2,2.9,-1.1)

CM	4.30 (24.6,10.8,1.3)	1.55 (66.9,23.4,20.1)	8.71 (-3.2,-10.7,-163.9)	1.78 (58.7,20.1,-12.8)	8.30 (10.2,2.9,-1.1)

MM	4.30 (24.6,10.8,1.3)	1.55 (66.9,23.4,20.1)	8.71 (-3.2,-10.7,-163.9)	1.79 (-10.6,-39.6,-91.3)	8.65 (10.2,2.9,-1.1)

C	3.89 (24.6,10.8,1.3)	1.30 (66.9,23.4,20.1)	8.98 (2.0,-7.2,-161.2)	1.97 (58.7,20.1,-12.8)	8.11 (10.2,2.9,-1.1)

Figure 1 shows the approximate position of these critical points in each component for the different criteria in the model of the restored tooth with ferrule. Big squares indicate the area for failure initiation of the system predicted by VM (in the crown) and by the rest of criteria (in dentine, in the cervical area under tensile stresses). Results show that the VM criterion predicts different failure positions in brittle components (core, root, cement) compared to the rest of the criteria. As noted before, the C criterion agrees with VM in the post and with other criteria for the brittle components with the exception of the crown, where MM and R predict the lowest SF near the load application area. A more detailed analysis of the results in the crown showed that the SF for this component is similar in the loading area and on the lingual side of the cervical area, where C and CM predict the failure, thereby indicating that both are critical areas for this component. Similar values of the SF were also observed with the VM criterion on the vestibular side of the cervical area and near the loading area.

**Figure 5:**
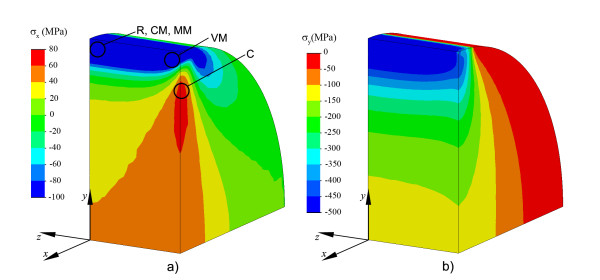
**Normal stress in the x-direction and y-direction in DTT**. Normal stress distribution in the x-direction (a) and y-direction (b) obtained in DTT for a disc thickness t = 3 mm, and location of predicted failure initiation areas for different failure criteria (a).

Figure 5 shows the stress distribution in the x and y directions in DTT for a disc thickness t = 3 mm. Stresses in the x-direction are mainly positive indicating tension in this direction, except in the area near to the loading points where compressive stresses are obtained. Tensile stress in a large area around the disc centre is near to 45 MPa, corresponding to analytical DTS (Eq. 10). However, tensile stresses greater than this value are obtained in the lateral faces of the cylindrical specimen for *z *= *t*/2. Stresses in the y-direction are compressive, and they are high compared to tensile stresses in the x-direction. Stresses in the z-direction are small except in the load application areas. Figure 5 also shows the areas where the different criteria predict failure initiation in the DTT. The C criterion predicts initiation in the area with greater tensile stress, while other criteria predict initiation near the loading area as a consequence of compressive stresses.

**Figure 6:**
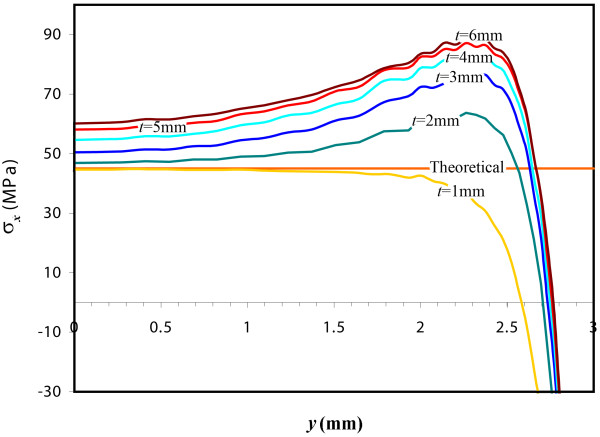
**Normal stress in the x-direction for DTT for the exterior diameter defined by z = t/2, x = 0**. Normal stress in the x-direction, *σ_x_*, along the line defined by *z = t/2, x = 0, y *= [0, *D*/2] for the six DTT models with thicknesses from 1 mm to 6 mm, and the theoretical result obtained with the plane stress hypothesis.

Figure 6 shows the effect of disc thickness in DTT on normal stress in the x-direction, *σ_x_*, along the line defined by *z = t/2, x = 0, y *= [0, *D*/2] for the 6 cylindrical specimens of different thicknesses and the theoretical result obtained with the plane stress hypothesis. As seen, the maximum value of *σ_x _*increases with disc thickness. As expected, the deviation from the plane stress theoretical constant value (45 MPa) increases with disc thickness. Actually, the deviation from the theoretical value is large, even for the thinner discs, when *y *tends to *D/2*.

**Figure 7:**
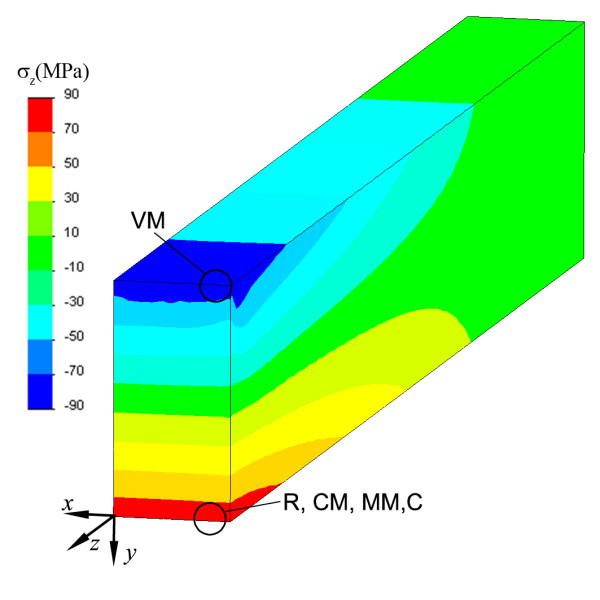
**Normal stress in the z-direction for FT**. Normal stress in the z-direction for the three-point flexural test simulation, and location of predicted failure initiation areas for different failure criteria.

Figure 7 shows normal stress in the z-direction for the three-point flexural test simulation. The stress distribution is typical of bending, with compression in the upper face and tension in the inferior face of the beam. The maximum tensile stress obtained is 85.0 MPa and shows a good agreement with the expected value (90 MPa). The maximum compressive stress is -178.7 MPa which is far from the theoretical value (-90 MPa) due to contact stresses. Normal stresses in other directions are small except near the loading point. Figure 7 also shows the areas where the different criteria predict failure initiation in the FT. All criteria predict failure in the tensile area of the central section except VM that predict failure in the compressive area.

The stress state (σ_3 _vs. σ_1_) of the critical points of the specimen for the DTT (*t *= 3 mm) and the FT predicted by each criterion compared with the failure lines for these criteria with TS = 100 MPa, CS = 300 MPa is shown in Figure 4. It can be observed that failure with FT, except for the VM criterion, is presented for a stress state with near uniaxial tensile stress in the inferior part of the specimen, whereas a more complex stress state is obtained with DTT regardless of the failure criterion considered.

## Discussion

The use of FEM to simulate dental restorations has increased considerably in the recent years, but a clear and correct procedure to interpret the results is needed to obtain valid conclusions. Complex stress states are present at the different points of the restoration requiring the use of failure criteria to determine whether these points will fail under this stress combination. Two main factors affect the correct interpretation of the results: the selection of the failure criterion and the values of TS and CS required for formulating this criterion.

Referring to the first factor, the results of the present work indicate that using the VM criterion is not adequate to interpret results in brittle components because this criterion is based only on TS. This conclusion is reinforced by the fact that, as has been shown in the results section for the restored tooth simulation, the VM criterion predicts failure at different locations compared to other criteria based on two properties (TS and CS) that are more adequate for brittle materials. A number of previous studies simulating endodontic restorations have interpreted the results based on the VM stress [4-6,8,12,13,16]. It can be concluded that some of these results should be taken with caution. The Christensen criterion has the advantage of managing both ductile and brittle materials with quite the same formulation, and the results of the present work indicate that the use of this criterion in the simulation of the restored tooth produces equivalent results to VM for ductile components and to CM and MM for brittle components. From the present results the authors recommend the use of this criterion for interpreting FEM results in dental restorations.

The prevalent use of the VM criterion in previous works is probably due to the fact that this is the usual criterion in the majority of engineering calculations and, also for this reason, it is incorporated as the default criterion in most commercial FE packages. The VM criterion makes it possible to perform a simple comparison between an equivalent stress and the tensile strength of the material, but it is only correct for ductile materials. The Christensen criterion has been presented quite recently in the literature [21], which explains why it is not present in commercial FE programs. For the same reason, it is absent in previous FE works on Endodontics. The use of this criterion involves some post-processing with the principal stresses and the TS and CS of the different materials. In any case, if it is not possible to use the C criterion, other long-established brittle criteria, such as CM or MM, are recommended instead of VM for brittle materials.

The second important factor for interpreting FEM results is the definition of material properties TS and CS. Obviously, a proper application of a failure criterion needs a sufficiently accurate value of TS and CS. Usually, obtaining an accurate value of CS is not problematic as it can be obtained from a simple compressive test. Dental materials manufacturers normally supply this data. In contrast, obtaining an accurate value of TS is not always easy. A direct tensile stress test is not suitable for brittle materials, and hence, tensile strength is commonly obtained from indirect tests like DTT or FT.

As for DTT two main assumptions must be made to use the DTS value obtained with Eq. 10 as an estimation of TS. First, it is necessary to assume a plane stress and point load to obtain Eq. 10 [36], and second it is necessary to assume failure initiation in the loading diameter and to neglect the effect of compressive stresses that are present in the disc.

The first assumption of the plane stress hypothesis will be satisfactory only for very thin specimen discs, *t/D*<< 1. Figure 6 shows that the maximum tensile stress value for each case goes away from the theoretical plane stress value as relation *t/D *increases. Only when *t *= 1 mm (*t/D = 0.17*) will the maximum tensile stress value obtained by FEA (44.83 MPa) agree with the theoretical value (45 MPa). However, if *t *= 3 mm (*t/D = 0.5)*, the maximum tensile stress (77.0 MPa) is far from the expected value and for thicker discs disagreement keeps growing. However, values of 0.5 for the thickness-to-diameter ratio are usual in the literature [23,26,27,37], probably to avoid buckling, and even greater values are used in some works, such as 0.8 [28] or 1.5 [29].

The second assumption is also difficult to justify. Figure 4 and Figure 5 show that the predicted failure initiation point is dependent on the failure criterion considered and that compressive stress is high for any of these possible failure points and cannot be neglected. For plane stress, compressive stress in the disc centre is equal to three times the tensile stress at this point, and this stress increases moving in the loading diameter towards the load application point, similar to result obtained with FEA, as shown in Figure 5. Neglecting this compressive stresses is equivalent to implicitly considering a failure criterion, not supported in the literature, given by max{*σ*_1_, *σ*_2_, *σ*_3_} ≤ *TS*. From the results shown in Figure 4, it can be concluded that failure in DTT is related to both tensile and compressive strengths, so the result of this test cannot be used to estimate TS. The situation is quite different for estimating TS from FS obtained with a FT. The FS value is obtained as the maximum tensile stress obtained in a flexural test for a simply supported beam (Eq. 11). To correctly apply Eq. 11, the cross section dimensions of the specimen must be much smaller than its longitudinal dimensions. The ISO 4049 standard defines appropriate test dimensions. FEA results for FT (see Figure 7) show good agreement between the *σ_z_*|_max _expected value (90MPa) and the FEA value (85.3MPa). Although high compression values appear in the contacts between the specimen and the anvils, that does not seem to be relevant for the flexural test. Figure 4 and Figure 7 show that the failure initiation point is in the part of the beam section under tensile stress, with a stress state near to uniaxial. Moreover, in this case all the failure criteria except Von Mises (not valid for brittle materials) predict failure at the same point.

Some works have reported differences between DTS and FS results for the same brittle material [23,29]. Generally, the FS value is greater than DTS. Our simulations confirm this observation. Figure 4 shows that, assuming the Christensen failure criterion, for a material with TS = 100 MPa and FS = 300 MPa, the DTS value will be lower than 45 MPa and the FS will be near 100 MPa.

From previous analyses, it can be concluded that FS is a good estimation for TS in brittle materials and can be used to interpret FEA results, whereas the DTS value is not a good option because is not clearly correlated to TS. Comparison of in vitro DTS of a material with tensile stresses experienced by a component of this material in a FEA, to assess a possible failure, as was done in previous studies [31], cannot be considered correct from the results of this study.

The results of the FE simulation of the endodontic restoration (Figure 1) also confirmed the problems with the use of the VM criterion in brittle materials, because this criterion predicts failure initiation points different to those observed with other criteria that are better suited for use with brittle materials. All the criteria other than the VM predicted the lowest SF in dentine, close to the interface with the crown, on the lingual side of the cervical area. This area is subject to high tensile stress, thus causing fracture of the dentine or the crown or loss of adhesion at the interface between crown and dentine, as has been observed in previous *in vitro *studies [40,41].

The present study has some limitations, as it considers isotropic linear materials in the restored tooth model. This assumption is common for most simulations, although some materials present some anisotropic behaviour, such as dentine or some composite materials with fillers. Moreover, some components, such as the PDL, introduce non-linear behaviour into the biomechanics of the restored tooth system and this effect is not included in our model, which is linear. Failure analysis for orthotropic or anisotropic materials is not dealt with in the present work. The failure analysis in these materials requires the use of more complex failure criteria [19] and material strength data in different directions are necessary.

## Conclusions

The following conclusions can be drawn from the present study related to the interpretation of finite element results in simulations of dental restorations:

In addressing the possible failure of the restored system, two main factors should be considered: the strength of the different materials involved (tensile strength and compressive strength) and the failure criterion to be used for each material.

The Von Mises criterion is a good option for ductile materials with equal tensile and compressive strength, but it fails with brittle materials. From the results of the present study we recommend the use of the Christensen criterion, which is valid for brittle materials and is coincident with Von Mises for ductile materials.

Flexural strength is a good estimation for tensile strength used in failure criteria. Diametral tensile strength should not be used in this context because failure under this test is related to both tensile and compressive strengths of the material.

## Competing interests

The authors declare that they have no competing interests.

## Authors' contributions

AP proposed the idea for this study and redacted most of the paper including the background, results and discussion. JLI participated in the design of the study and carried out the FEAs for DTS and FS. CG participated in the revision of literature and the development of the FE model of the endodontic restoration. All authors revised the final manuscript.
