# Correction to: Interpreting finite element results for brittle materials in endodontic restorations

**DOI:** 10.1186/s12893-023-02116-1

**Published:** 2023-08-14

**Authors:** Antonio Pérez-González, José L Iserte-Vilar, Carmen González-Lluch

**Affiliations:** Department of Mechanical Engineering and Construction, Universitat Jaume I, Castellón de la Plana, Spain

Following publication of the original article [1], in this article the Eq. 7, has been updated as shown below:

$$S{F_{C\_duc}} = \frac{{TS}}{{\frac{{\left( {k - 1} \right)}}{{2k}} \cdot {I_1} + \sqrt {{{\left( {\frac{{k - 1}}{{2k}}} \right)}^2} \cdot I_1^2 - \frac{1}{k} \cdot 3 \cdot {J_2}} }}$$

